# Do obese patients benefit from isolated aortic valve replacement through a partial upper sternotomy?

**DOI:** 10.1186/s13019-022-01926-3

**Published:** 2022-08-03

**Authors:** Xian-Biao Xie, Xiao-Fu Dai, Zhi-Huang Qiu, De-Bin Jiang, Qing-Song Wu, Yi Dong, Liang-Wan Chen

**Affiliations:** 1 Department of Cardiovascular Surgery, Union Hospital, Fujian Medical University, Fuzhou, 350001 Fujian China; 2 Key Laboratory of Cardio-Thoracic Surgery, Fujian Medical University, Fujian Province University, Fuzhou, Fujian China; 3 Fujian Provincial Special Reserve Talents Laboratory, Fuzhou, Fujian China

**Keywords:** Aortic valve replacement, Obesity, Partial upper sternotomy

## Abstract

**Objective:**

Controversial opinions exist for aortic valve replacement (AVR) through partial upper sternotomy in obese patients. Moreover, this study sought to investigate the potential clinical advantage of partial upper sternotomy aortic valve replacement (mini-AVR) over conventional full sternotomy aortic valve replacement (con-AVR) in obese patients.

**Methods:**

This was a retrospective and observational study. From January 2015 to December 2020, a total of 184 obese [body mass index (BMI) ≥ 30 kg  m^2^] patients undergoing isolated primary AVR were included: 98 patients underwent conventional full sternotomy, and 86 patients underwent partial upper sternotomy. Propensity score (PS) matching was applied to eliminate the bassline imbalances in the mini-AVR and the con-AVR groups.

**Results:**

After one-to-one propensity score matching, two groups of 60 patients were obtained. No in-hospital death occurred in the two groups. In addition, cardiopulmonary bypass time and total operative time were similar across the 2 groups, but the aortic cross-clamp time was significantly shorter in the con-AVR group (*P* = .0.022). The amount of mediastinal drainage at 48 h after surgery (*P* =  0.018) and postoperative blood transfusions (*P* =  0.014) were significantly lower in the mini-AVR group. There was no difference in ventilation time (*P* = .0.145), but a shorter intensive care unit stay time (*P* =  0.021) in the mini-AVR group.

**Conclusion:**

This study demonstrates that aortic valve replacement through a mini-AVR in obese patients is a safe and effective procedure. It outperformed con-AVR in terms of blood loss, blood product transfusion, and ICU stay.

## Introduction

Currently, in light of the rapid development of science, people's accelerated pace of work and life, the reduction of physical activity and unhealthy diet, people have a higher nutritional intake than that which they need given their levels of caloric exertion, and obesity is becoming an increasingly hefty global health problem [1]. The proportion of obese patients [body mass index (BMI) ≥ 30 kg m^2^] in cardiac surgery continues to rise. Obesity does not increase cardiac surgical mortality, but does increase the incidence of postoperative ventilator use, acute kidney injury, and poor incision healing [2]. Since the 1990s, AVR through partial upper sternotomy (mini-AVR) has been proven to be safe, without increasing in-hospital mortality and postoperative complications [3]. Many articles have compared the advantages and disadvantages of aortic valve replacement via a mini-AVR versus conventional full sternotomy (con-AVR) [4–6]. However, these articles consist of comparative studies of minimal invasive versus conventional aortic valve replacement in the general population, while a previous report has specifically described aortic valve replacement (AVR) through partial upper sternotomy in obese patients. Obese patients often also suffer from obstructive sleep apnea–hypopnea syndrome, hypertension, or Type 2 diabetes; these illnesses tend to lead to respiratory complications, heart failure and sternum infection [7]. Therefore, we believe that aortic valve replacement through a mini-AVR is of great benefit to obese patients.

The aim of this study was to investigate whether AVR through a mini-AVR is feasible and superior to a con-AVR in obese patients.

## Material and methods

### Patients

**Fig. 1:**
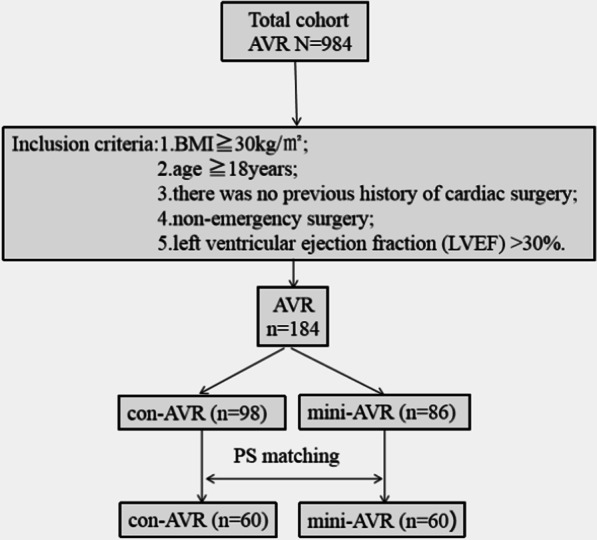
A CONSORT type diagram of the patients with aortic valve disease were underwent isolated AVR. AVR, Aortic valve replacement; PS, propensity score

This is a retrospective study, which was approved by our institution’s review board. It prospectively collected data from 984 patients with aortic valve disease who underwent isolated AVR in our department between January 2015 and December 2020. Of these, a total of 184 patients met the following criteria: (1) preoperative BMI ≥ 30 kg m^2^; (2) age ≥ 18 years; (3) no previous cardiac surgery; (4) non-emergency operation; and (5) left ventricular ejection fraction > 30%. Of the 184 patients, 98 underwent surgery through a con-AVR, and 86 underwent through a mini-AVR. All operations were performed by a senior surgeon in our department. Because of the differences in the preoperative characteristics and to avoid selection bias, data analysis was performed using a propensity score matching, with 60 matched patients in each group (Fig. 1).

### Surgical technique for mini-AVR

All patients were operated on in a supine position and under static aspiration compound anesthesia. A midline skin incision approximately 8 to 10 cm in length was performed from the manubriosternal junction to the third or fourth (BMI > 40 kg/m^2^)intercostal space (ICS). A “J-shaped” sternotomy exiting through the right third or fourth ICS was used. It was important to avoid damaging the right mammary artery. Next, the sternum was opened with a small sternum opener, the pericardium was opened and pulled on, and the ascending aorta, superior vena cava, and right atrial appendage were exposed. Following full systemic heparinization, cardiopulmonary bypass was established using direct central aortic and right atrium cannulation despite the limited space available. A left ventricular vent was placed through the right upper pulmonary vein. Mild hypothermia (systemic temperature of 32 °C) was induced consistently. The aortic cross-clamp was applied and hypothermic 4 °C blood cardioplegia was antegradely infused into the aortic root to stop the heart and in case of aortic regurgitation directly into the coronary ostia. The operative field was insufflated with carbon dioxide during the entire operation. Following the stopping of the heart, the diseased valve and surrounding calcium were removed, after which a standard biological or mechanical prosthesis was inserted.

### Statistical analysis

Propensity score matching analysis was performed to reduce potential selection bias with the potential confounders between the con-AVR and the mini-AVR groups using calipers of width equal to 0.2 of the SD of the logit of the propensity score. We examined the similarity between the con-AVR and the mini-AVR groups by calculating standardized differences for each of the baseline variables. All standardized differences for each baseline variable were < 0.2 (20%).

All statistical analyses were performed using Statistical Package for the Social Sciences (IBM Corp. Released 2015. IBM SPSS Statistics for Windows, Version 23.0. Armonk, NY: IBM Corp). Continuous variables were tested for normal distribution using a Kolmogorov–Smirnov test. Continuous variables with a normal distribution are expressed as mean ± standard deviation (SD) and compared using a Student’s t-test; otherwise, they are expressed as median (interquartile range [IQR]) and compared with a Mann–Whitney U test. Categorical data were analyzed using the chi-square test or Fisher’s exact test as appropriate. *P*-value < 0.05 was considered statistically significant.

## Results

Between January 2015 and December 2020, 984 patients with aortic valve disease underwent isolated AVR in our department. A total of 184 individuals were eligible for enrollment. Of these, 98 individuals were treated with con-AVR and 86 individuals were treated with mini-AVR. Following propensity score matching, 60 matched pairs were included in the analysis with a 60:60 (mini-AVR:con-AVR) ratio. These patients were matched according to age, BMI, hemoglobin (Hb) and hematocrit (HCT) and were found to be comparable between the two groups. Thereafter, there was no longer any significant difference between the 2 groups for any covariate (Table 1).

**Table 1:** Baseline characteristics before and after propensity score matching

Variable	All patients			Propensity matched patients		
	con-AVR (n = 98)	mini-AVR (n = 86)	*P*-value	con-AVR (n = 60)	mini-AVR (n = 60)	*P*-value
Age, mean ± SD	57.8 ± 12.3	62.9 ± 10.7	< 0.001	60.1 ± 10.9	60.6 ± 11.3	.878
Male [n (%)]	58(59.2)	46(53.5)	.343	32 (67)	28 (61)	.465
BMI, kg/m^2^, mean ± SD	35.5 ± 4.2	38.3 ± 5.3	< 0.001	35.9 ± 4.5	34.9 ± 5.1	.762
BMI > 40 kg/m^2^ [n (%)]	8 (8.1)	6 (6.9)	.731	6 (10.0)	5 (8.3)	.751
EuroScore, median (IQR)	7 (3–12)	7 (3–11)	.871	7 (3–11)	7 (3–11)	1.000
Ejection fraction (EF), % median [IQR]	60 (50–65)	59 (49–64)	.256	60 (52–65)	60 (55–66)	.576
History of AF [n (%)]	9 (9.1)	9 (10.4)	.965	7 (11.7)	6 (10.0)	1.000
Diabetes mellitus [n (%)]	8 (8.1)	6 (6.9)	.731	6 (10.0)	5 (8.3)	.751
Hypertension [n (%)]	62 (63.2)	52 (60.4)	.564	46 (76.7)	43 (71.7)	.531
Cerebrovascular disease [n (%)]	5 (5.1)	5 (5.8)	.909	3 (5.0)	2 (3.3)	1.000
COPD [n (%)]	5 (5.1)	4 (4.6)	1	2 (3.2)	2 (3.2)	1.000
Smoking history [n (%)]	34 (34.7)	29 (33.7)	.850	21 (35.0)	20 (33.3)	.847
Hb (mg/dl), mean ± SD	12.22 ± 1.35	13.68 ± 1.68	< 0.001	12.48 ± 1.65	13.12 ± 1.55	.534
HCT (%), mean ± SD	38.32 ± 4.68	41.32 ± 3.68	< 0.001	39.82 ± 3.78	40.32 ± 3.65	.337

*BMI* body mass index, *SD* standard deviation; *IQR* interquartile range, *AF* atrial fibrillation, *COPD* chronic obstructive pulmonary disease, *Hb* haemoglobin, *HCT* haematocrit

There were no patients who needed to be converted from mini-AVR to con-AVR. No significant between-group differences in cardiopulmonary bypass time, total operative time, or diameter of implanted aortic valve prosthesis were found but the aortic cross-clamp time was significantly shorter in the con-AVR group (56.38 ± 12.60 min vs 62.32 ± 14.45 min; *P* = 0.022). The percentage of patients who were implanted any type of aortic valve prosthesis was similar across the 2 groups (Table 2).

**Table 2:** Operative data after propensity score matching

	con-AVR (n = 60)	mini-AVR (n = 60)	*P*-value
Cross-clamp time,min, mean ± SD	56.38 ± 12.60	62.32 ± 14.45	0.022
CPB time,min, mean ± SD	85.52 ± 15.35	90.68 ± 16.54	0.745
Total operative time,min, mean ± SD	173.32 ± 41.61	176.38 ± 38.45	0.461
Prosthetic valves			0.706
Mechanical, n (%)	39(65)	36(60)	
Biologic, n (%)	21(35)	24(40)	
Diameter of implanted prosthesis,mm, mean ± SD	23.34 ± 2.55	22.8 ± 2.24	0.213

*AVR* aortic valve replacement, *CPB* cardiopulmonary bypass, *SD* standard deviation

Postoperative results following propensity score matching in both groups are shown in Table 3. There were no in-hospital deaths across the 2 groups. The mean quantity of mediastinal drainage volume at 48 h following surgery (150.0 ml [IQR, 80.0–800.0 ml] vs. 220.0 ml [IQR, 100.0–600.0 ml]; *P* = 0.018) and red blood cell transfusion (130.0 ml [IQR, 0–800.0 ml] vs. 200.0 ml [IQR, 0–600.0 ml]; *P* = 0.014) were significantly lower in the mini-AVR group as compared to the con-AVR group. No patient needed reopening for bleeding in the con-AVR group; however, one patient was reopened for bleeding from the right mammary artery in the mini-AVR group. The mean time in the mechanical ventilator was (5.0 h [IQR, 2.0–49.0 h] vs. 6.0 h [IQR, 2.0–125 h]; *P* = 0.145). This was similar in the mini-AVR group and the con-AVR group. The incidences of low cardiac output syndrome, respiratory insufficiency, and renal insufficiency were similar across the 2 groups. There was no significant difference in the rates of reintubation, sternum refixation, and wound infection between the mini-AVR and con-AVR groups. The mean ICU stay was shorter in the mini-AVR group as compared to the con-AVR group (15.0 h [IQR, 8.0–75.0 h] vs. 19.0 h [IQR, 10.0–168 h]; *P* = 0.021). The median duration of hospital stay in the mini-AVR group was 10.0 days [IQR, 5.0–18 days], which was not significantly different from con-AVR group (11.0 days [IQR, 5.0–26.0 days]; *P* = 0.258).

**Table 3:** Postoperative results after propensity score matching

Variable	con-AVR (n = 60)	mini-AVR (n = 60)	*P*-value
Mediastinal drainage, mL/48 h, median (IQR)	220.0 (100.0, 600.0)	150.0 (80.0, 800.0)	0.018
Red blood cell transfusion, mL, median (IQR)	200.0 (0, 600.0)	130.0 (0, 800.0)	0.014
Reexploration due to bleeding, n (%)	0	1 (1.7)	1.000
Ventilation time (h), median (IQR)	6 (2, 125)	5 (2, 49)	0.145
Reintubation (%), n (%)	2 (3.3)	0	0.476
Low cardiac output syndrome, n (%)	1 (1.7)	2 (3.3)	1.000
Respiratory insufficiency, n (%)	9 (15)	8 (13.3)	1.000
Renal insufficiency			1.000
Without dialysis,n (%)	4 (6.7)	4 (6.7)	
With dialysis,n (%)	2 (3.3)	3 (5.0)	
Wound infection (%)	0	1 (1.7)	1.000
Sternum refixation, n (%)	0	2 (3.3)	1.000
ICU stay,h, median (IQR)	19 (10, 168)	15 (8, 75)	0.021
Hospital stay, d, median (IQR)	11.0(5.0, 26)	10.0(5.0, 18)	0.258
In-hospital mortality, n (%)	0	0	1.000

*AVR* aortic valve replacement, *SD* standard deviation, *IQR* interquartile range, *ICU* Intensive care unit

## Discussion

Our short-term data suggests that mini-AVR is as safe and effective as con-AVR in obese patients. There were no differences in cardiopulmonary bypass time, total operative time, or diameter of implanted aortic valve prosthesis across the 2 groups. The incidences of reintubation, sternum refixation, wound infection, low cardiac output syndrome, respiratory insufficiency, and renal insufficiency were not different between mini-AVR and con-AVR patients, and the length of hospital stay was also similar. Most importantly, our results showed that the postoperative mediastinal drainage and red blood cell transfusion were significantly lower in the mini-AVR group, as well as a shortened ICU stay. However, in this study, one patient in the mini-AVR group needed to be reopened due to bleeding because their right mammary artery was damaged, thereby serving as are minder to be careful not to damage the right mammary artery during the operation.

Minimally invasive AVR through upper partial sternotomy was introduced by Svensson in 1997 [8]. Currently, upper partial sternotomy has been widely used in the context of aortic valve surgery, aortic root surgery, and even acute type A aortic dissection surgery [9]. AVR through partial upper sternotomy can yield excellent results in terms of postoperative pain and reduction of hospitalization, ventilation times, occurrence of renal failure, and need for blood transfusion [3, 10, 11]. In recent years, although transcatheter AVR has developed rapidly and surgical indications have been further expanded, it is still mainly performed in medium- and high-risk patients [12, 13]. AVR through a right anterolateral minimally invasive incision often requires femoral artery and vein intubation and may increase neurological complications [14]; therefore, partial upper sternotomy is the most common incision for minimally invasive AVR in our center. The worldwide prevalence of obesity is at its highest level ever recorded and continues to increase [15]. In China, more than 50% of adults are overweight or obese, with overweight and obesity rates of 34.3% and 16.4%, respectively, making the Chinese population the largest obese population worldwide [16]. The proportion of obese patients in cardiac surgery continues to grow. Brinkman et al. [17] believe that the chest wall of obese patients is hypertrophic and that surgical field exposure is harder in heart surgery as such; therefore, it should be carefully considered in the context of heart surgery through partial upper sternotomy in obese patients. Several studies have revealed that the mini-AVR group has the same postoperative mortality and major complication rates as the con-AVR group in obese patients [4, 18]. Our study further supports this idea.

Mini-AVR increases cardiopulmonary bypass time, total operative time and aortic cross-clamp time due to exposure difficulties [19, 20]. Our study further showed that only the aortic cross-clamp time was increased and the cardiopulmonary bypass time and total operative time were slightly longer; however, the difference was not statistically significant. This may be attributed to two different reasons. First, when a patient was extremely obese (BMI > 40 kg/m^2^), the incision was extended to the fourth intercostal space. The exposure provided by this procedure was excellent. Second, due to diminished bleeding and shorter surgical incision, the time for hemostasis and chest closure was shortened. Therefore, in extremely obese patients (BMI > 40 kg/m^2^), we can extend the incision to the fourth intercostal level. This can provide a better exposure of the operational area, and this may also lead to better sternal stability compared to full sternotomy, thereby stabilizing the thorax while still ensuring the advantages of the partial upper sternotomy.

Some scholars believe that obese patients suffer from excessive hypertrophy of the chest wall and poor compliance, and are prone to postoperative sternum shaking and surgical incision infection and other complications [2, 21]. While these complications are not life-threatening, they can prolong hospital stay and increase the risk of acquiring a nosocomial infection. In our study, two patients required sternum refixation, as did one patient with wound infection in the mini-AVR group; however, no patient in the con-AVR group did. This result is contrary to our subjective clinical impression, possibly showing that the pathogenesis of sternum shivering or infection is multifaceted and that partial upper sternotomy is not enough to reduce the probability of this complication occurring.

Excess adipose tissue in the abdomen and around the chest wall of obese patients presses the chest and immerses the breathing muscles, limiting the motility of the chest and the diaphragm in a way that is more pronounced during supine sleep [7]. Therefore, lung capacity and functional residual volume are reduced and breathing becomes shallow and fast, resulting in an increase in dead cavity ventilation, effective alveolar ventilation, and a maximum amount of independent ventilation. Therefore, obese patients are obviously affected as regards their respiratory function, which often leads to a prolonged ICU stay. In addition, the incidence of tracheal intubation and reintubation following postoperative extraction is higher in obese patients as compared to general patients, both of which increase the use of hospital resources [22]. A partial upper sternotomy can reduce postoperative pain and the psychological burden of patients. It can also encourage patients to get out of bed early after surgery and accelerate the recovery of respiratory function and physical function. It is more aligned with the rapid rehabilitation concept of modern medicine [23, 24]. Therefore, we believe that obese patients can benefit more from a partial upper sternotomy than can non-obese patients.

## Limitations

We acknowledge there are limitations to this study. First, this is a retrospective study, and, although we applied a propensity matching analysis, we still cannot completely overlook that the presence of unknown confounding variables might affect the observations. Second, in this study, we defined obesity only as a BMI of ≥  30 kg m^2^, because BMI calculations use overall body weight, without reflecting the distribution of body fat and the proportion of fat in one’s body. Therefore, people with a normal weight but excess fat may be overlooked, while those with higher muscle tissue content may be misdiagnosed. the study included a rather small number of patients.

## Conclusions

Aortic valve replacement through a mini-AVR in obese patients is a feasible technique. As compared to con-AVR, significant benefits of blood loss, blood product transfusion, and ICU stay were clearly observed.

## Data Availability

Data sharing not applicable to this article as no data sets were generated or analyzed during the current study.

## Abbreviations

AVR: Aortic valve replacement
BMI: Body mass index
ICS: Intercostal space
ICU: Intensive care unit
CPB: Cardiopulmonary bypass
IQR: Interquartile range
SD: Standard deviation
PS: Propensity score
Hb: Haemoglobin
HCT: Haematocrit
